# Diagnostic accuracy of a modularized, virtual-reality-based automated pupillometer for detection of relative afferent pupillary defect in unilateral optic neuropathies

**DOI:** 10.3389/fopht.2024.1396511

**Published:** 2024-09-03

**Authors:** Rahul Negi, Manasa Kalivemula, Karan Bisht, Manjushree Bhate, Virender Sachdeva, Shrikant R. Bharadwaj

**Affiliations:** ^1^ Brien Holden Institute of Optometry and Vision Sciences, L V Prasad Eye Institute, Hyderabad, Telangana, India; ^2^ Center for Technology Innovation, L V Prasad Eye Institute, Hyderabad, Telangana, India; ^3^ Child Sight Institute, L V Prasad Eye Institute, Hyderabad, Telangana, India; ^4^ Nimmagadda Prasad Children’s Eye Care Centre, L V Prasad Eye Institute, Visakhapatnam, Andhra Pradesh, India; ^5^ Prof. Brien Holden Eye Research Centre, Hyderabad Eye Research Foundation, L V Prasad Eye Institute, Hyderabad, Telangana, India

**Keywords:** diagnostic accuracy, infrared, neuro-ophthalmic pathology, edge detection, swinging flashlight test, virtual reality, pupillometry

## Abstract

**Purpose:**

To describe the construction and diagnostic accuracy of a modularized, virtual reality (VR)-based, pupillometer for detecting relative afferent pupillary defect (RAPD) in unilateral optic neuropathies, vis-à-vis, clinical grading by experienced neuro-ophthalmologists.

**Methods:**

Protocols for the swinging flashlight test and pupillary light response analysis used in a previous stand-alone pupillometer was integrated into the hardware of a Pico Neo 2 Eye^®^ VR headset with built-in eye tracker. Each eye of 77 cases (mean ± 1SD age: 39.1 ± 14.9yrs) and 77 age-similar controls were stimulated independently thrice for 1sec at 125lux light intensity, followed by 3sec of darkness. RAPD was quantified as the ratio of the direct reflex of the stronger to the weaker eye. Device performance was evaluated using standard ROC analysis.

**Results:**

The median (25th – 75th quartiles) pupil constriction of the affected eye of cases was 38% (17 – 23%) smaller than their fellow eye (p<0.001), compared to an interocular difference of +/-6% (3 – 15%) in controls. The sensitivity of RAPD detection was 78.5% for the entire dataset and it improved to 85.1% when the physiological asymmetries in the bilateral pupillary miosis were accounted for. Specificity and the area under ROC curve remained between 81 – 96.3% across all analyses.

**Conclusions:**

RAPD may be successfully quantified in unilateral neuro-ophthalmic pathology using a VR-technology-based modularized pupillometer. Such an objective estimation of RAPD provides immunity against biases and variability in the clinical grading, overall enhancing its value for clinical decision making.

## Introduction

The swinging flashlight test remains the primary technique for the assessment of relative afferent pupillary defect (RAPD) in patients with unilateral neuro-ophthalmic pathology (1–3). Classically, in this test, light from a pen-torch is shone alternatively in each eye for 3 seconds to elicit the pupillary light reflex. The eye generating the weaker direct reflex is suspected to have a RAPD and its intensity is graded according to the classification described by Bell (1, 4). However, significant errors might arise in the estimation of RAPD from the variability in executing the technique, the examiners’ ability to pick interocular difference in pupillary constriction and in judgment of RAPD grade by the examiner (3, 5–11). For instance, the detection of RAPD in this test is critically dependent on the clinician’s sensitivity to appreciate interocular differences in the pupillary responses (3, 5–11). The test procedure also varies significantly across clinicians, with light sources that elicit the pupillary light reflex varying in their intensity, spectral composition, retinal irradiance and in the duration and frequency of stimulation of two eyes (3, 5–11). All these variables interact in a complex manner to influence the swinging flashlight test outcomes, often times obfuscating the RAPD estimates on the patient. While some of these procedural variabilities may be addressed by adhering to the guidelines put forth by Kelbsch et al. (1), the inherently qualitative nature of the test will continue to add variability to the estimations of RAPD using this test.

Video-based pupillometry has long been recognized as a technology that helps improve the diagnostic accuracy of the swinging flashlight test, enabling evidence-based management of patients with neuro-ophthalmic dysfunction (9, 12–21). Accordingly, several commercial devices are now available for objective pupillometry and quantification of the RAPD severity [e.g., NeurOptics^®^ RAPiDo™ Neuroptics Inc, Irvine, USA (16); EyeKinetix^®^, Konan Medical Inc, Irvine, USA (22, 23); BulbiCAM^®^, Bulbitech AS, Dybdahls, Norway; Pupil+^®^, L V Prasad Eye Institute, Hyderabad, India (9)]. The cost-utility ratio of these stand-alone pupillometers may be improved by modularizing and integrating their functionality into existing technology, such as virtual reality (VR) displays (12, 17). Many forms of conventional assessments and therapy have their VR display equivalents in today’s clinical practice [e.g., visual functions assessment, disease monitoring and game-based therapies for amblyopia and binocular vision anomalies] (24–28). The integration of eye trackers into VR headsets further widens their scope for an assessment of oculomotor behavior using these devices (29, 30). VR displays may thus be readily utilized to present calibrated light stimuli to elicit pupillary responses that can then be measured using the integrated eye tracker. In fact, two such attempts have been made in the recent past, wherein commercially available VR-headsets with built-in eye trackers (Fove DK0, Fove Inc., Japan and HTC Vive Pro Eye, HTC Corporation, Taiwan) were adapted for the assessment of RAPD (12, 17). The study by Bruegger et al. is of particular interest to the present study, for this study evaluated the diagnostic accuracy of this technology in patients with unilateral optic neuropathy who manifested varying grades of RAPD (12). They reported a sensitivity and specificity of 90.2% and 82.2%, respectively, in detecting RAPD, vis-à-vis, gold-standard clinical assessments (12). The study by Sarkar et al. evaluated the test-retest reliability of such a technology in estimating RAPD in otherwise normal individuals (17). Their results showed moderate to good reliability of the RAPD measurements using VR-based technology, with the Bland-Altman type analysis showing test-retest ranges from 0.02 to 0.07 units across different protocols and devices (17).

The Pupil+^®^ pupillometer, a stand-alone device for the video-based assessment of RAPD, has been developed at the L V Prasad Eye Institute, India, the affiliating body of the study authors. The Pupil+^®^ pupillometer is described in detail by Negi et al. (9). This device was recently upgraded by modularizing the original design and integrating it into a commercially-available Pico Neo 2 Eye^®^ VR headset (Pico^®^, California, USA) with built-in eye tracking capabilities (Tobii AB^®^, Stockholm, Sweden). The primary aim of the current study is to describe the diagnostic accuracy of the upgraded device by measuring the RAPD scores of healthy controls and cases with unilateral neuro-ophthalmic pathology, vis-à-vis, judgments of experienced neuro-ophthalmologists. The overarching goal is to replicate the technology reported recently by Bruegger et al. (12) and Sarkar et al. (17) and determine the utility of such a technological upgrade in identifying pupillary manifestations of unilateral neuro-ophthalmic pathologies in a tertiary-level eye hospital that manages a very diverse clinical population.

## Methods

### Development of the modularized pupillometer

**Figure 1:**
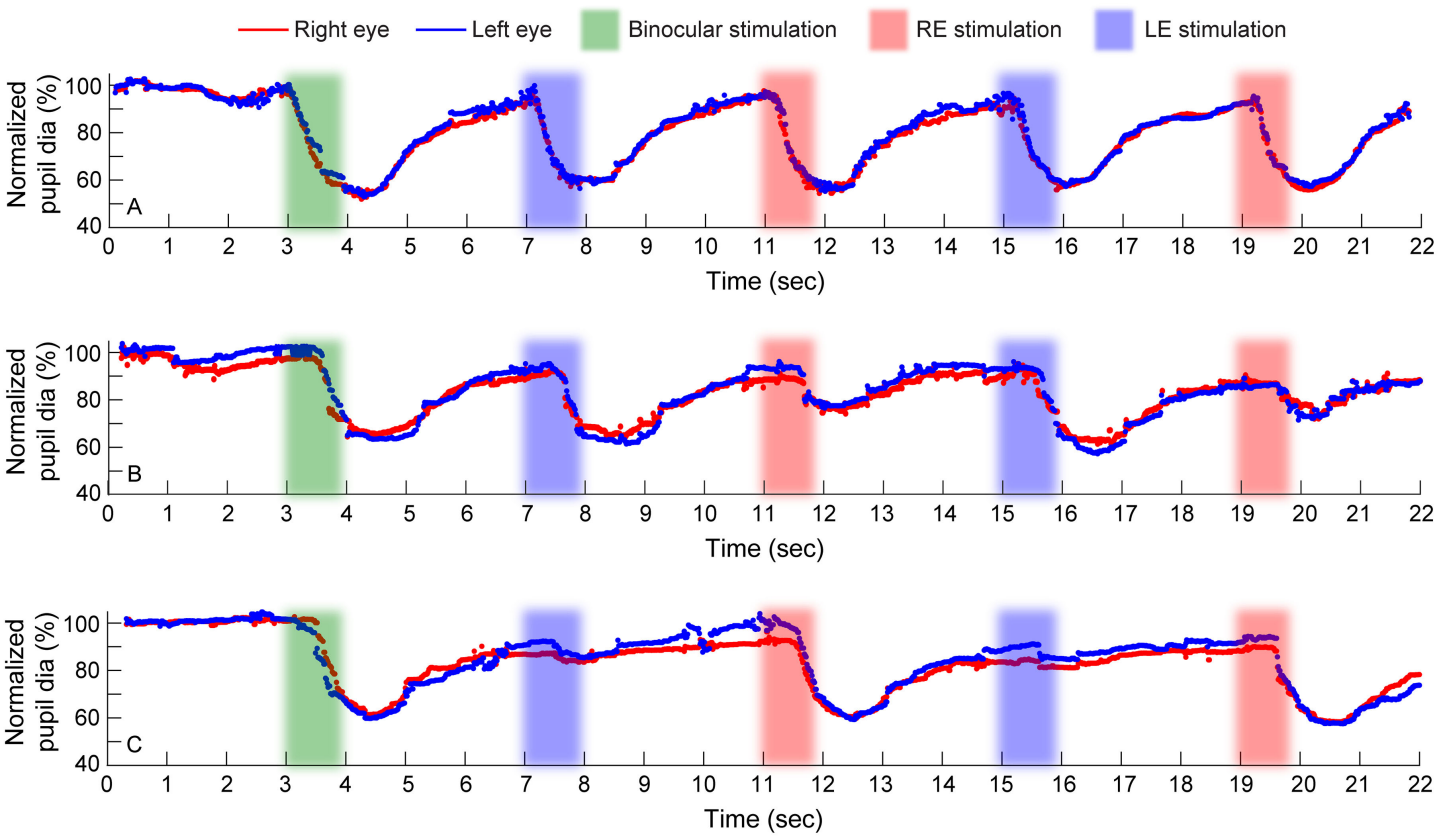
Raw traces of normalized pupillary diameters of the right and left eyes plotted as a function of time for a representative control **(A)**, a case with right eye RAPD **(B)** and another case with left eye RAPD **(C)**. Data from two of the three cycles of light stimulation are shown here. The raw traces are plotted as percentage change in the pupil constriction relative to the baseline dark-adapted state. Stimulation of both eyes at the beginning of the trial and stimulation of the right and left eyes independently are indicated by differently colored bands. The time epoch before the green band represents the dark adaptation provided to the subject.

The Pupil+ pupillometer was upgraded by completely replacing its hardware with the Pico Neo 2 device. Following the participant’s alignment with the device, the swinging flashlight test protocol was implemented in alignment with the standards for clinical RAPD testing (1). The paradigm started with an initial 5 seconds-long period of complete darkness for both pupils to reach a steady baseline state, followed by a 1-second-long light pulse to both eyes simultaneously and 3-seconds of darkness (**Figure 1**). This was followed by three cycles of independent light stimulation of the two eyes, with each cycle consisting of a 1-second-long pulse of 125lux to one eye followed by 3-seconds of darkness (**Figure 1**) (9). This temporal sequence of light simulation followed the recommendations of the standards for assessment of RAPD by Kelbsch et al. (1). The intensity of light stimulation was chosen following Negi et al. (9) who showed the 125lux of light intensity to be the most optimal at eliciting a RAPD in patients with unilateral neuro-ophthalmic pathology – intensities lower than this led to poor signal to noise ratio of pupillary light reflex and those higher than this led to an attenuation of the RAPD in majority of the patients in their study (9). The left was always stimulated first followed by the same profile for the right eye (**Figure 1**). The VR display was calibrated using a lux meter from 8 cm to ensure that consistent light pulses were delivered to each during the entire measurement protocol. This calibration was checked once in a week to ensure consistency of light intensity across testing. The pupil diameter of each eye was determined using the built-in eye tracker at 90 frames per second. The software module containing the swinging flashlight test protocol and RAPD score calculator was both developed on the Unity platform (Unity Technologies, San Francisco, USA) and operated out of a cloud-based server in the upgraded device.

### Assessment of the diagnostic accuracy of the upgraded device

#### Subjects

The protocol for this prospective, case-control study adhered to the tenets of the Declaration of Helsinki and was approved by the institutional review boards of the Hyderabad and Vishakhapatnam campuses of the L V Prasad Eye Institute, India. All cases and controls signed a written informed consent form prior to study participation. For participants <18 years of age, an assent form from the child and consent form from the parents/guardians was taken. Cases with clinically diagnosed unilateral optic neuropathies in either eye were recruited from the patient pool of the institute. Patients presenting with binocular disease, those showing greater than 30 prism diopters (*ΔD)* of tropia, frequent blinking and those who expressed reluctance to participate were excluded from the study. Children <7 years of age were also excluded owing to the snug placement of the VR device on their heads and their narrow interpupillary distance sometimes posing a challenge for accurate pupil measurements. The presence of RAPD was confirmed by the treating neuro-ophthalmologists using the swinging flashlight test. Clinical diagnosis of the neuro-ophthalmic pathology was based on the standard practice guidelines that involve a detailed history and ocular examination, pupil assessment and the gradation of RAPD using the Bell’s grading system (4), fundus photographs, losses in visual acuity, contrast sensitivity and color vision, MRI findings, and other ocular and systemic observations. Clinical management of these patients also followed standard guidelines, with no impact of the present study outcomes on their management. Data from visually healthy controls were also recruited for the study from the student/staff pool of the institute. All controls had best-corrected visual acuity of 20/25 or better, no known deficiencies in color vision, and were deemed to be free of RAPD following assessment by an experienced clinician using the standard clinical swinging flashlight test.

### Procedure and calculation of the RAPD score

Once recruited into the study, the participants wore the VR headset, and it was adjusted to snugly fit their head. Participants were then instructed not to move their eyes and refrain from blinking as much as possible during the test. The swinging flashlight test protocol was then initiated, and the output was saved for offline analysis. The test protocol alone took approximately 30sec to complete while the entire test, starting from the wearing of the VR headset to test completion lasted for approximately 5min. The raw data of the pupillary diameters obtained from the eye tracker were plotted as a function of time and smoothed using a 100-samples running average filter. Data points corresponding to blinks or large eye movements were excluded by applying a lower and upper cut-off of pupil diameters (<1mm to >9mm) and these were replaced by interpolating the raw data using the Piecewise Cubic Hermite Interpolating Polynomial (PCHIP) interpolation algorithm. This algorithm typically failed for missing data points for >1sec (i.e., 90 data points in sequence). Such traces were deemed to have poor data quality and explored from the analysis. The test was repeated on the participant in such instances. Once the measurement was deemed appropriate, the algorithm evaluated the consensual pupil responses for any sign of efferent pathway defects. Such defects were flagged and the algorithm did not proceed with the calculation of RAPD scores. Once the efferent pupillary defects were ruled out, the RAPD score was determined by assessing the percentage change in pupil constriction between the non-affected and affected eyes. Thus, the data of all the study participants reported here are free from efferent pupillary pathway abnormalities. All these signal processing techniques were implemented using Matlab (R2016a, Natick, USA). The RAPD score calculation involved deriving a ratio of the affected eye’s percentage change by the non-affected eye’s percentage change such that the resulting RAPD score was always greater than unity. This rendered the RAPD score distribution a one-tailed one, facilitating easy calculation of the diagnostic accuracy of the present device. This approach is slightly different from the classic way of deriving the RAPD score in which the ratio is always obtained by dividing the right eye’s response over the left’s eye response or vice versa, irrespective of which eye generated the larger response. In this scheme, RAPD scores will form a two-tailed distribution, with ratios greater than unity indicating the eye in the denominator having the weaker response and ratios lesser than unity indicating the eye in the numerator having the weaker response.

### Data analyses

Data analyses were performed using Microsoft Excel (Microsoft Corporation, Redmond, USA), and Matlab. Kolmogorov–Smirnov test indicated that the direct and consensual pupillary light responses were normally distributed, and hence parametric statistics were used to compare the difference in means between the groups for direct and consensual pupillary light responses. The RAPD scores, on the other hand, were not normally distributed and hence non-parametric statistics were used for comparison. The diagnostic accuracy of the device was determined using a receiver operating characteristic (ROC) analysis from which outcome variables of area under the curve, sensitivity, specificity, accuracy, and precision were computed. The reliability of the RAPD score as a binary classifier of the presence/absence of RAPD was determined using the Matthews correlation coefficient (MCC) (31). For all these analyses, the RAPD detected by the experienced neuro-ophthalmologist was considered as the gold-standard, even while acknowledging that this grading may be subject to significant intra- and inter-examiner variability (5, 7, 11). The RAPD grades were classified in accordance with the Bell’s classification wherein mild RAPD corresponded to grades 1 and 2 in the classification scheme and ≥moderate RAPD corresponded to grades 3 – 5 in the classification scheme (4).

## Results

### Subject demographics

Seventy-seven cases (age range: 8 – 75yrs; 49 male) with unilateral neuro-ophthalmic pathology and an equal number of age-similar controls (9 – 65yrs; 47 male) participated in the study. The cases were diagnosed to have a variety of neuro-ophthalmic pathology, including optic neuritis, optic atrophy, retrobulbar neuritis, traumatic optic neuropathy, and non-arteritic anterior ischemic optic neuropathy, impacting either the right eye (n=34) or the left eye (n=43). The mean ( ± 1SD) best-corrected high contrast visual acuity of cases and controls were 1.09 ± 1.10 logMAR units and 0.00 ± 0.00logMAR units, respectively (p<0.001).

### Pupillary responses

**Figure 1** plots the raw data of pupillary responses of representative subjects to the repeated sequence of the right and the left eye stimulation using the VR-based pupillometer. Robust direct pupillary miosis was observed for both eyes in controls (mean ± 1SD: 38.76 ± 2.10%) (**Figure 1A**), while the direct reflex of the affected eye of cases was attenuated relative to the fellow eye (**Figures 1B, C**). For the case with right eye RAPD, the right eye direct reflex (12.24 ± 1.24%) was smaller than the left eye (26.74 ± 3.97%) (**Figure 1B**), and conversely in patients with left eye RAPD (right eye direct reflex: 34.43 ± 0.35%; left eye direct reflex: 6.63 ± 0.92%) (**Figure 1C**). Consensual pupillary reflexes from these eyes were robust in all subjects (**Figure 1**).

**Figure 2:**
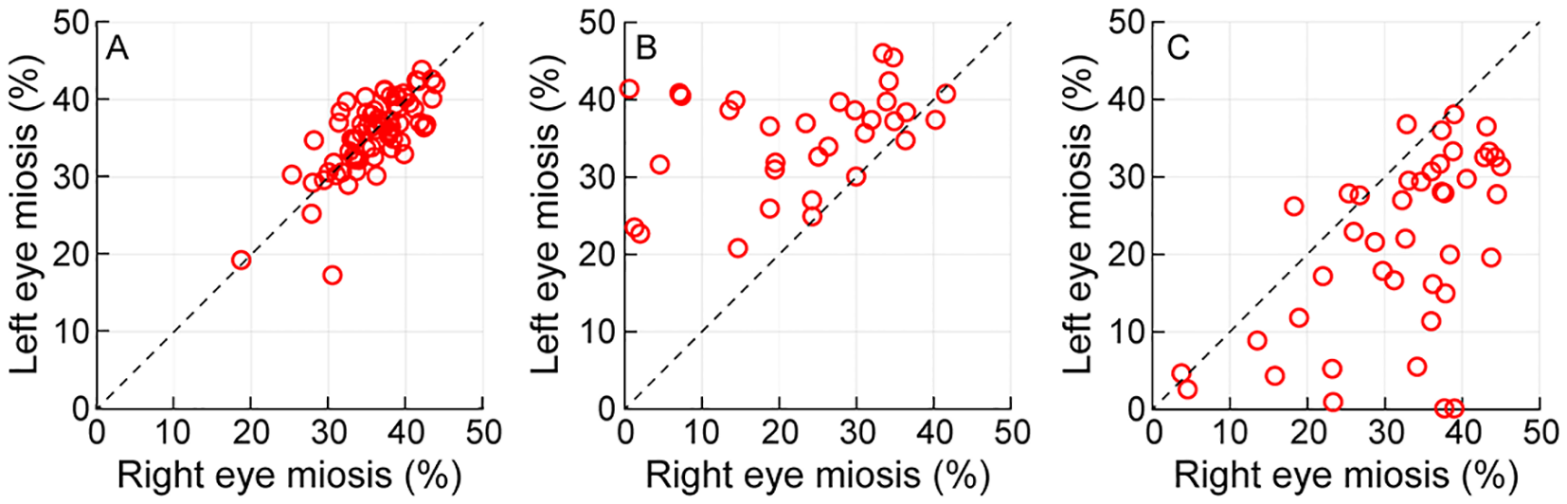
Scatter diagram of the percentage change in the direct pupillary constriction of the left and right eyes plotted against each other for controls **(A)**, cases with right eye RAPD **(B)** and cases with left eye RAPD **(C)**. The diagonal line in each panel indicates the line of equal pupillary response magnitude.

**Figure 2** shows a scatter diagram of the right eye and the left eye’s direct reflexes plotted against each other for all controls (Panel A) and cases with right eye RAPD (Panel B) and left eye RAPD (Panel C) that participated in the study. The data lay along the 1:1 line in controls, indicating similar magnitudes of direct pupillary reflexes in both eyes [mean ( ± 1SD) right eye: 36.09 ± 4.59%; left eye: 35.65 ± 4.78%; p=0.83] (**Figure 2A**). The direct reflexes of cases with right eye RAPD were significantly smaller in the right eye (23.25 ± 11.82%) relative to the left eye (36.67 ± 10.91%) (p<0.001) and this was reflected in the data points lying above the 1:1 line in **Figure 2B**. The trend reversed in the left eye RAPD cohort, with the mean ( ± 1SD) direct pupil constriction of the left eye (21.55 ± 11.46%) being significantly smaller than that of the right eye (32.40 ± 10.36%) (p<0.001) (**Figure 2C**). The standard deviation of pupillary constriction was larger in cases compared to controls, reflecting larger inter subject variability of the data in the former than the latter cohort (**Figure 2**). This perhaps arose from pooling of all the data of cases into one group, irrespective of the severity of their neuro-ophthalmic pathology.

### RAPD scores

**Figure 3:**
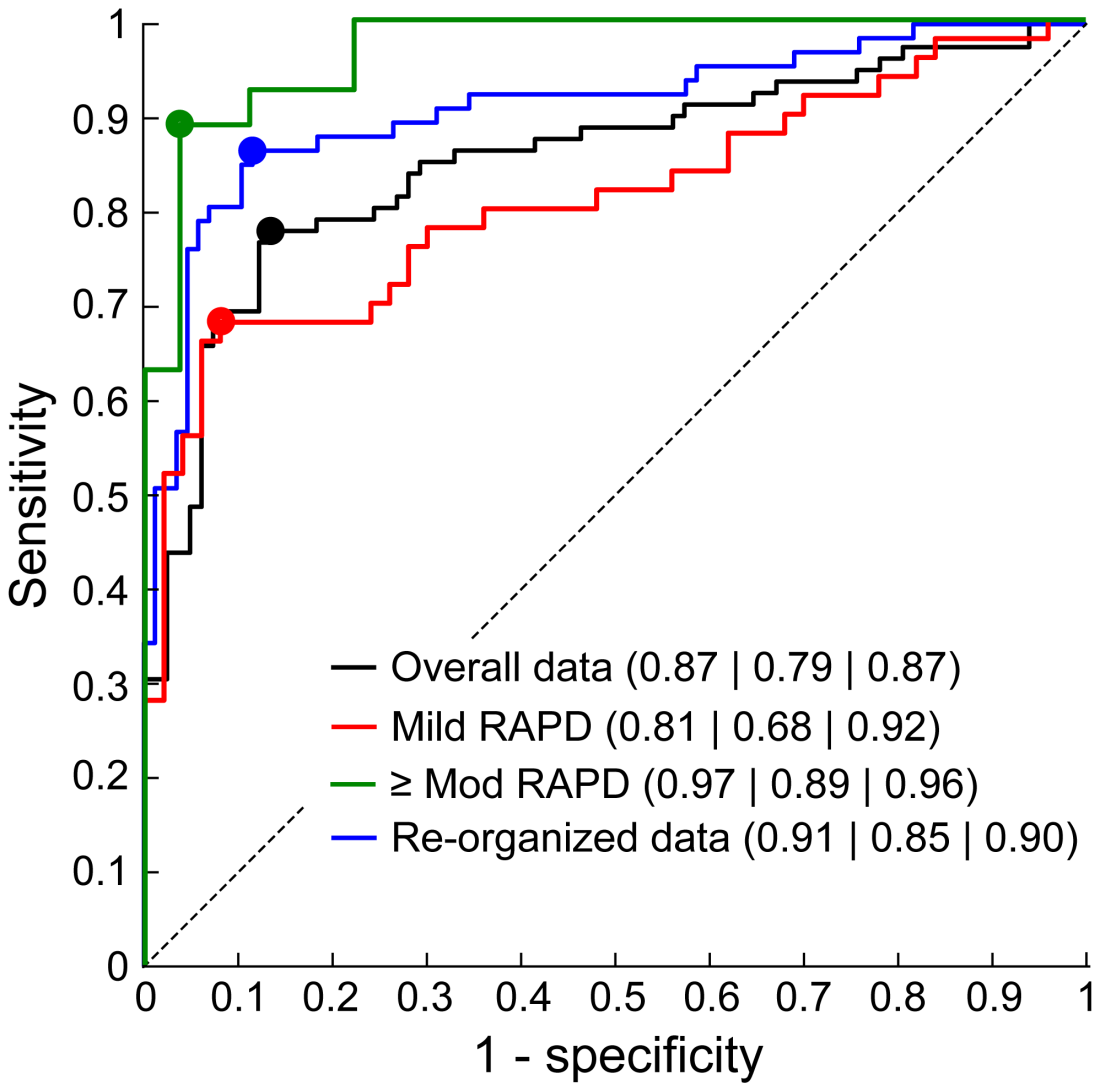
Receiver operating characteristics (ROC) curves of the modularized pupillometer along with the key diagnostic indicators [area under ROC (AUROC)SensitivitySpecificity] plotted for the original dataset, data with only mild severity of RAPD and only moderate or higher severities of RAPD and with the data of controls re-organized to account for physiological variability in pupillomotor output in the two eyes (green trace; see text for details). The circles overlying each ROC curve corresponds to the best sensitivity and 1-specificity values obtained from the Youden J’s index. See **Table 1** for additional details.

The median (25^th^ – 75^th^ quartiles) RAPD score of cases with RAPD [1.38 (1.17 – 2.23)] was significantly higher than controls [1.06 (1.03 – 1.11)] (Mann-Whitney U test; p=0.001). The score of cases of with right [1.31 (1.12 – 1.872)] and left [1.34 (1.17 – 1.62)] eye RAPD were not statistically significantly different from each other (p=0.66). The ROC curve demonstrating this pupillometer’s ability to detect RAPD, vis-à-vis, clinical judgments is shown in **Figure 3** and **Table 1**. While this ROC analysis did show potential for the pupillometer to differentiate cases from controls, the device incorrectly labeled 13% of controls as having RAPD (87% specificity) and missed RAPD in 21.5% of cases (78.5% sensitivity) (**Table 1**). The following analyses were undertaken to investigate the potential reason for these sensitivity/specificity values.

**Table 1:** Diagnostic accuracy of the objective pupillometer in diagnosing RAPD, vis-à-vis, clinical judgments of the study participants.

	Youden’s cut-off	Area under ROC curve	Sensitivity (%)	Specificity (%)	Accuracy (%)	Precision (%)	MCC
**Overall** (n=77)	1.17	0.87	78.5	87.0	82.7	85.7	0.66
**Mild RAPD** (n=50)	1.17	0.81	68.0	92.0	80.0	89.5	0.62
**≥Moderate RAPD** (n=27)	1.50	0.97	89.1	96.3	92.6	96.0	0.85
**Re-organized** (n=77)	1.17	0.91	85.1	89.7	87.7	85.3	0.75

These outcome variables were obtained following a standard ROC analysis. Data of ROC curves obtained using the overall data (second row), only for cases with mild RAPD (third row), only for cases with moderate or higher severities of RAPD (fourth row) and controls data re-organized (last row) are shown in this table. MCC: Matthews Correlation Coefficient (31).

First, even though experienced and overtly unbiased, the gold standard judgments by the clinician may be subject to some degree of bias and variability (5, 7, 11). For instance, the clinical judgment could be biased in favor of the presence of RAPD if the associated findings on the patient signal the presence of a neuro-ophthalmic pathology (e.g., color vision abnormality, visual field defects, optic nerve evaluation). In reality, this may just be a physiological difference in the pupillomotor output of the two eyes, as demonstrated by Wilhelm et al. (32) in a minority of otherwise healthy individuals. This clinical over-diagnosis of RAPD may appear as false negatives in objective pupillometry, thereby reducing the measurement sensitivity in the ROC analysis (**Table 1**). Measurements of specificity, on the other hand, may remain unaffected because of a low apriori in detecting pupillary abnormality in healthy controls. Such a bias may manifest more in cases with mild rather than moderate or severe neuro-ophthalmic disease. This hypothesis was tested by dividing the cases into those with Grades I and II RAPD (mild RAPD; n=50 out of 77) and Grade III or higher RAPD (moderate RAPD; n=22 of 77), based on their clinical records (4). Controls data were randomly sampled to match the number of cases for this analysis. In support of the hypothesis, the reconstructed ROC curve for the mild cases showed lower area under the curve and sensitivity, relative to the curve or cases with ≥moderate RAPD (**Table 1** and **Figure 3**). The specificity remained largely unaffected (**Table 1** and **Figure 3**).

As a second confirmatory analysis, the RAPD scores of cases lower than the 25th quartile of this cohort (i.e., RAPD scores ≤1.17) were identified (n=20 of 77) and equally distributed between the controls and cases in random order to overcome for the effect of the clinical over-diagnosis. This cut-off value was in line with Wilhelm et al.’s observation of the pupillomotor output varying up to 20% between the two eyes of healthy individuals (32). The re-plotted ROC curve showed a significant improvement in the area under the ROC curve and the sensitivity values, relative to the original classification (**Table 1** and **Figure 3**). As before, the specificity did not alter much with this re-classification (~2%; **Table 1** and **Figure 3**). Repeating the ROC analysis by changing the random allocation of the 20 cases changed the area under the ROC curve by no more than 1% of what is reported in **Table 1**.

## Discussion

The study presents two important outcomes. First, on the technology front, the study shows that a stand-alone objective pupillometer may be successfully modularized and its hardware may be integrated into a commercial VR-based system with built-in eye tracking capability. The software implementing the swinging flashlight test protocol can then be used to obtain reliable estimates of RAPD in patients with unilateral neuro-ophthalmic pathology (**Figures 1**, **2**). This capability is line with the recent report by Bruegger et al. (12) who had integrated a similar RAPD testing protocol on the Fove DK0 VR headset with a built-in binocular eye tracker. The modularization of the pupillometry hardware and software reported here is evaluated presently only for the Pico Neo series of VR headsets with built-in eye tracking capability. In theory, the software (RAPD stimulation protocol and pupillary response evaluation) should also be compatible with other VR headsets available in the market. However, this needs explicit testing and calibration for the programming regime, frame rate of eye tracking and other technical specifications may vary significantly across VR headsets. Second, on the technique front, the results show that the inherent biases and subjective variability in the routine clinical assessment of RAPD may be overcome using objective pupillometers, including the one described in this study (**Table 1** and **Figure 3**). The study implications are discussed below.

The technological advancement reported in this study effectively removes the need for using stand-alone devices for the assessment of pupillary light reflexes in the clinic and it also make such assessments agnostic to the hardware used. VR displays already show significant promise in their ability to replace physical tests that form an integral part of the comprehensive eye examination into electronic equivalents that can be easily administered and the outcomes accessed remotely through cloud-based servers (24–27). Several eye exam procedures may be administrated using VR displays while the patient waits to meet an eye care professional, thus de-clogging over-stretched tertiary care centers. Like other branches of medicine (33, 34), conventional tele-eye health initiatives may also be augmented by administering many of these tests at the door-step of the patient, thus overcoming the accessibility barrier to quality eye care. The present study indicates that pupillary assessments can now be added to the list of tests that may be effectively administered using VR displays. Moving forward, such a technology may be adopted as part of the routine eye examination in primary to tertiary level eye clinics. The objective nature of this technology, combined with the ease of data analytics, may allow a more rigorous longitudinal follow-up of patients with neuro-ophthalmic pathology and assess the impact of medical/surgical interventions on their recovery [e.g., optic neuritis, multiple sclerosis (35, 36)]. Additional protocols for the assessment of pupillary health, including an assessment of pupil shape, dynamics (latency, reaction time, peak velocity, etc), properties of the post-illumination pupillary response and chromatic pupillometry may also be incorporated into VR headsets in the future (1, 37, 38).

Present-day VR technology for objective pupillometry is not without its limitations. This technology generated error-free estimates of the pupillary light reflex only on those without any significant eye deviations (< 30ΔD of tropia). VR technology may be challenging in infants and toddlers with narrow interpupillary distances and/or who may feel uncomfortable with the immersive nature of this headset – this limits its use for assessing RAPD in children with developmental pathologies like amblyopia (39, 40). Similarly, this technology will be challenging to use in individuals who cannot be instructed to wear a VR headset or keep their eyes still during the measurements (e.g., individuals in the neurodiverse spectrum or patients in critical care units). In its present state, the accommodative demand also remains fixed in VR displays (41) and hence its influence on the pupillary light reflex or an assessment of the pupillary near-responses is not immediately possible. Physiologically, the near miosis is an integral component of pupillary assessment and it reflects the health of the near-triad (accommodation, binocular vergence and pupillary miosis) (42, 43), as regulated by the III nerve complex in the brain stem (44, 45). The pupillary near reflex also plays an important role in identifying chronic neuro-ophthalmic pathologies [e.g., light-near dissociation in Adie’s tonic pupils (46, 47)] (48). This limitation may be overcome in the future by using augmented reality displays that allow overlay of the virtual reality stimuli with the real-world scene (49).

Despite its limitations (3, 5–11), the qualitative outcome of the swinging flashlight test remains the gold-standard for the detection of RAPD in the clinic. The ROC curves shown in **Figure 3** of this study indicates that such a comparison may lead to undesirably lower values of sensitivity with which an objective pupillometer may differentiate cases with unilateral neuro-ophthalmic pathology from controls. Specificity may not be affected as much, as shown from its stability across the different ways of constructing the ROC curves in **Table 1**, **Figure 3**. The ROC values reported here are comparable to those obtained recently with a VR-based pupillometer (12) and with two commercially available stand-alone pupillometers. Bruegger et al. reported a sensitivity, specificity and accuracy of 82.2%, 87.5% and 84.% in their VR-based pupillometer using patients with varying severities of RAPD, as assessed using the swinging flashlight test with neural density filters (12). The NeurOptics^®^ RAPiDo™ has a reported sensitivity and specificity of 89% and 91.7%, respectively (16), while the EyeKinetix^®^ objective pupillometer has a reported sensitivity and specificity of 82% and 94% for the detection of RAPD (21). The relatively lower sensitivities in all these devices may arise from the over-diagnosis of clinical RAPD, as reported in this study. Fixing this issue by accounting for the physiological variability of pupillomotor output in the two eyes may significantly improve the ability of these devices to differentiate pupil abnormalities in cases from controls (**Table 1** and **Figure 3**). Despite this correction, the sensitivity did not reach 100%, indicating that the measures of RAPD may be inherently limited in their ability to pick subtle neuro-ophthalmic pathology. Such an inability may arise from the pupillary hippus masking subtle changes in the pupillary light reflex (50) or from the light intensity that was used to stimulate the pupils being non-optimal for every subject that participated in this study (9). The intensity of 125 lux was chosen based on the average response of patients with unilateral neuro-ophthalmic pathology reported by Negi et al. (9). However, that study also showed sizeable intersubject variability in the pattern of pupillary response to varying light intensity that is not straightforward to factor into the pupillary light stimulation algorithm used for estimating RAPD. Additionally, the clinical grading of RAPD was not always performed at this light intensity, adding to the variability observed in this study. These limitations notwithstanding, objective assessment of RAPD is certainly a step-up to the presently used clinical testing methods. Moving forward, the RAPD scores obtained from objective pupillometers such as the one describe here may be used in conjunction with the larger neuro-ophthalmic battery, including optic color vision loss, abnormal visually evoked potentials and other imaging modalities like magnetic resonance imaging to gain additional insights into the disease pathophysiology and treatment outcomes.

## Data Availability

The raw data supporting the conclusions of this article will be made available by the authors, without undue reservation.
